# *Anisakis* Sensitization in the Croatian fish processing workers: Behavioral instead of occupational risk factors?

**DOI:** 10.1371/journal.pntd.0008038

**Published:** 2020-01-27

**Authors:** Ana Jerončić, Diana Nonković, Anamarija Vrbatović, Jerko Hrabar, Ivana Bušelić, Victoria Martínez-Sernández, Santiago A. Lojo Rocamonde, Florencio M. Ubeira, Sonja Jaman, Esma Čečuk Jeličić, Marco Amati, Maria Angeles Gomez Morales, Boris Lukšić, Ivona Mladineo

**Affiliations:** 1 University of Split, School of Medicine, Split, Croatia; 2 Teaching Institute of Public Health, County of Dalmatia, Split, Croatia; 3 Institute of Oceanography and Fisheries, Split, Croatia; 4 Faculty of Pharmacy, University of Santiago de Compostela, Santiago de Compostela, Spain; 5 Servicio de Análisis Clínicos, Complejo Hospitalario Universitario de Santiago de Compostela (CHUS), Santiago de Compostela, Spain; 6 Clinical Hospital Center Split, Split, Croatia; 7 Istituto Superiore di Sanità, Roma, Italy; NIH-NIRT-ICER, INDIA

## Abstract

We undertook the first study systematically evaluating the risk of *Anisakis*-sensitization in Croatian fish-processing workers and potential genetic susceptibility to anisakiasis. Anti-*Anisakis* IgE seroprevalence and risk factors for 600 employees of Croatian fish processing facilities and 466 blood donor controls, were assessed by indirect ELISA targeted with: recombinant Ani s 1 and Ani s 7 allergens, an *Anisakis* crude extract, the commercial ImmunoCAP kit, and questionnaires. Genetic susceptibility to anisakiasis was evaluated by genotypisation of human leukocytes alleles (HLA). Anti-*Anisakis* seropositive and a fraction of negative subjects were also assessed by ELISA and Western Blot (WB) for IgG seroprevalence to *Trichinella* spp. Overall, the observed anti-*Anisakis* seroprevalence inferred by indirect ELISA was significantly higher in fish processing workers (1.8%, 95% CI 0.9–3.3%) compared to the controls (0%, 0–0.8%). Seven out of 11 Ani s 1 and Ani s 7-positives and none of selected 65 negative sera, tested positive on whole-*Anisakis* extract (ImmunoCAP), whereas *Anisakis* crude extract ELISA detected 3.9% (2.4–6.0%) seropositives in fish processing workers, three (14%) of which showed IgE reactivity to milk proteins. The highest risk associated with *Anisakis*-sensitization among workers was fishing in the free time, rather than any of attributes related to the occupational exposure. Although no association was observed between anti-*Anisakis* seropositivity and wearing gloves or protective goggles, the majority of workers (92%) wore protective gloves, minimizing the risk for *Anisakis* sensitization via skin contact. Six HLA alleles within DRB1 gene were significantly associated with seropositivity under dominant, allelic or recessive models. All sera confirmed negative for anti-*Trichinella* spp. IgG. The study exhaustively covered almost all marine fish processing workers in Croatia, reflecting real-time *Anisakis* sensitization status within the industry, already under the influence of wide array of allergens.

## Author summary

Anisakiasis is a human disease caused by ingestion of live *Anisakis* spp. larvae by raw seafood. Fish processing and aquaculture workers (approximately 60 million people world-wide) are at occupational risk of becoming sensitized to *Anisakis*. This is the first study systematically evaluating the risk of *Anisakis*-sensitization in Croatian fish-processing workers and potential genetic susceptibility to anisakiasis in the analysed population. Observed seroprevalence was significantly higher in fish processing workers (1.8%) compared to the controls (0%). Surprisingly, the highest risk associated with *Anisakis*-sensitization among workers was fishing in their free time, rather than any of attributes related to the occupational exposure. While no association was observed between anti-*Anisakis* seropositivity and wearing gloves or protective goggles, the majority of workers (92%) wore protective gloves, minimizing the risk for *Anisakis* sensitization via skin contact. Additionally, seroprevalence to another important food-borne helminth, *Trichinella* spp. assessed in all *Anisakis*-positive subjects showed to be negative. Almost all marine fish processing workers in Croatia were part of this research, reflecting real-time *Anisakis*-sensitization status within the industry. This sets a baseline for the future tracking of sensitization in the sector already under the influence of wide array of allergens.

## Introduction

Anisakiasis is a human disease contracted by consumption of inadequately thermally processed seafood harboring infective third-stage larvae of the nematode genus *Anisakis*. It is an important emerging foodborne disease, recently ranked as fifth in the European risk ranking and the second of 24 foodborne parasitoses with the highest "increasing illness potential" [1]. Four specific forms are recognized; gastric, intestinal, ectopic and gastro-allergic [2], but the frequent misdiagnoses and underreporting of cases leads to the biased data interpretation and a speculative incidence. Likewise, one of the latest sources estimated 20 cases per year at European level [3], while a recently developed quantitative risk model suggested only in Spain between 7700 and 8320 of cases annually [4]. In light of these findings, Moneo et al. [5] suggested a fifth anisakiasis form important from public health prospective that encompasses sensitized asymptomatic patients, otherwise healthy individuals with a high anti-*Anisakis* IgE titer that potentially underwent a subclinical or undiagnosed gastric anisakiasis without allergic symptoms. Due to the lack of clinical symptoms, this form of anisakiasis, which may account for more than 12% prevalence in some populations even using component-resolved diagnosis [6,7], is mainly detected in the course of epidemiological studies [8,9]. Also, since the presence of anti-*Anisakis* IgE antibodies in sensitized patients is detectable in serum during many years, which in some cases could be boosted by continuous ingestion of *Anisakis* allergens with food [10], it is not possible to know the time of primo-infection in such individuals.

It was reported that allergic reactions to *Anisakis* allergens can also occur without a previous infection either by inhalation or direct contact in the domestic or occupational environment [11]. In particular, contact and air borne exposures were mainly reported in occupational settings of fishery and aquaculture workers, cooks, fishmongers and anglers [12,13]. In such cases, patients exhibit symptoms of rhino-conjunctivitis, asthma, anaphylaxis and dermatitis [14,15], which frequently lead to increased incapacity and absenteeism from work [16]. Albeit it is less frequently reported compared to orally-exposed *Anisakis* allergy, non-oral exposure could have a significant impact within public health domain, as according to FAO almost 60 million people are engaged in primary sector of fisheries and aquaculture [17].

Croatian fish processing industry is a valuable sector for both national and international market, with an annual revenue of 112 MU$, encompassing circa 1150 employees in freshwater, marine fish, crustacean and bivalve processing sector [18]. It is important to highlight that these data encompass both the freshwater and marine fish, crustacean and bivalve processing sectors, since no official statistics in Croatia differentiate between specific sector types. However, our estimation is that at least two-thirds of total production is effectuated by marine fish processing facilities.

In a previous study we estimated that the overall *Anisakis* seroprevalence in Croatian apparently healthy population is 2% [9]. Now we aimed to investigate: i) the IgE seroprevalence to *Anisakis* allergens (Ani s 1 and Ani s 7 allergens, *Anisakis* crude extract allergens, allergens included in the ImmunoCAP kit) in workers that are professionally exposed to contact, air and food-borne in the fish processing industry, and ii) the potential risk factors for *Anisakis*-sensitization, including genetic susceptibility to anisakiasis by genotyping of human leukocytes alleles (HLA). Complementarily, we assessed i) the risk of suffering cutaneous and/ or respiratory allergic symptoms in fish-processing workers, and ii) the IgG sensitization to another foodborne helminth *Trichinella* spp., in a subsample of subjects including those testing positive for anisakiasis.

## Materials and methods

### Ethics approval and consent to participate

This research was approved by the Ethics Committee of the Teaching Institute of Public Health, County of Split-Dalmatia, class: 500-01/14-01/1, no: 2181-103-01-14. Written informed consent was obtained from all individual adult participants included in the study.

### Participants and study design

**Fig 1:**
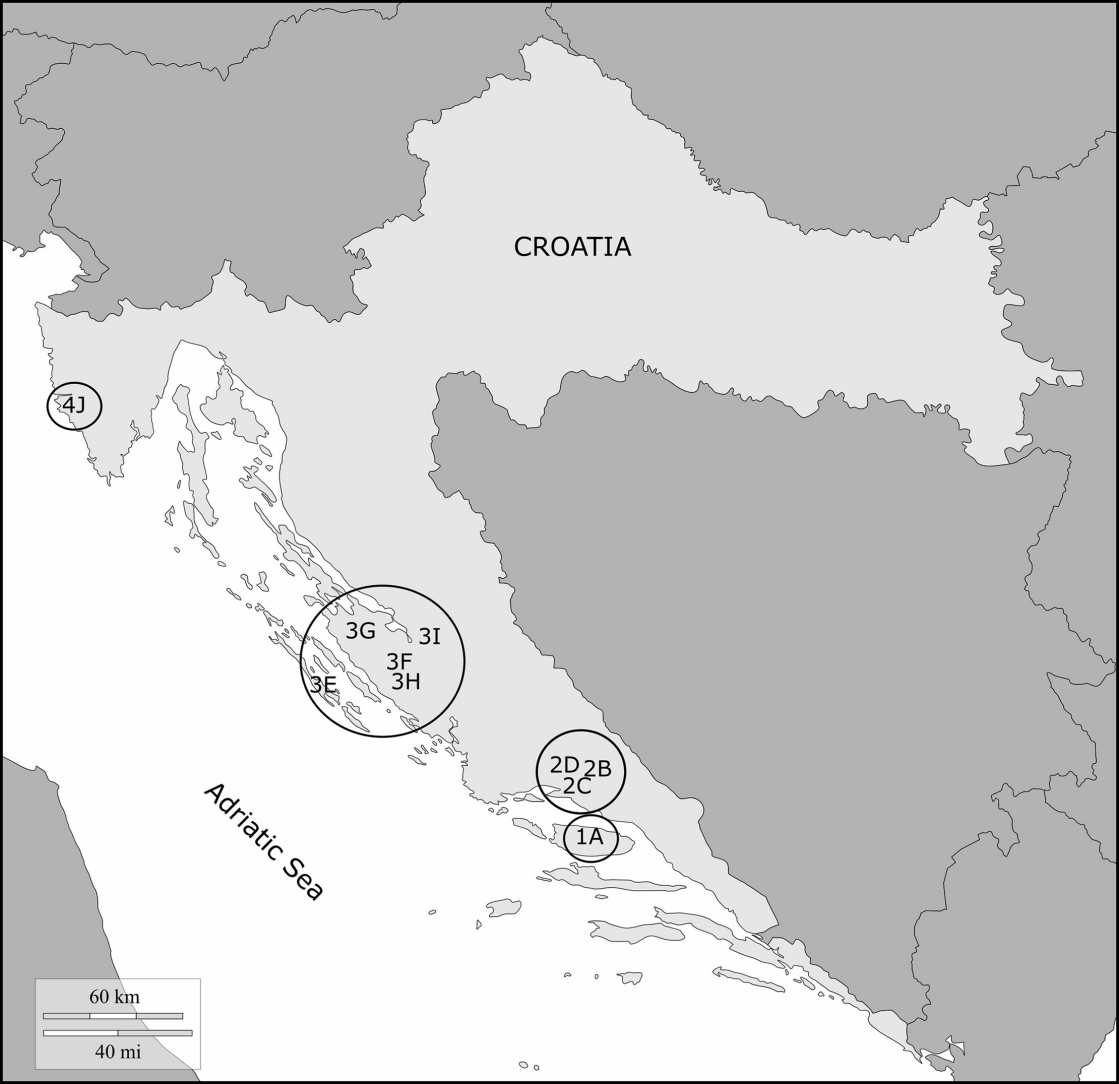
Geographic map of Croatia where facilities included in the seroepidemiological study of anti-*Anisakis* sensitization are shown as circles with a code. 1A: Centre 1 (Brac 43°19′N 16°38′E), facility A Sardina; 2B: Centre 2 (Sinj, 43°42′N 16°38′E), facility B Conex; 2C: Centre 2, facility C Felicita; 2D: Centre 2, facility D Trenton; 3E: Centre 3 (Zadar, 44°6′51''N 15°13′40''E), facility E Sali Mardesic; 3F: Centre 3, facility F Omega Benkovac; 3G: Centre 3, facility G Mislov; 3H: Centre 3, facility H Ostrea; 3I: Centre 3, facility I Noclerius; 4J: Centre 4 (Rovinj, 45°04′59''N 13°38′402''E), facility J Mirna. The map was adapted from freely available figure on https://d-maps.com/pays.php?num_pay=319&lang=en.

This was a multicenter, cross-sectional study encompassing all marine fish processing facilities in Croatia, conducted from December 2015 till December 2016 (Fig 1). All employed workers were invited to join the study, and 600 (78%) were sampled. Also were sampled 446 apparently healthy Croatian working-age adults not associated with the industry who were sampled from the same underlying population (controls). A STROBE checklist is provided in S1 Checklist. More details on centers and facilities are presented in S1 Table. Detailed information on the study design and sampling protocol is given in S1 Data. A sample questionnaire is provided in S2 Data, while the distribution of responses to selected questionnaire' items is shown in S1 Fig.

Briefly, among the subjects who finished filling the questionnaire (n = 563; 94%), two groups were established: Group A (n = 371; 67%) that comprised workers not self-reporting allergic diseases, and Group B (n = 192; 34%) that self-reported allergic diseases. According to the tasks carried out in the facilities, non-allergic and allergic workers were then subdivided in three subgroups each, respectively (see Table 1): subgroups A1 and B1 (n = 268 and 147, respectively) included subjects working in direct contact with fish; A2 and B2 (n = 39 and 22, respectively) those working in indirect contact with fish; A3 and B3 (n = 64 and 23, respectively) subjects working in tasks with no evident contact with fish. In addition, workers included in allergic group (Group B) were further subclassified depending on the type of allergic disease they reported (Table 1) into: subgroup that included workers reporting cutaneous allergy symptoms (BA1; n = 47), workers reporting manifestations of rhino-conjunctivitis (BA2; n = 56), those reporting respiratory symptoms (BA3; n = 47), and those that included workers reporting symptoms encompassed in two or more of the above categories (BA4; n = 42).

**Table 1:** Number of the fish-processing workers grouped by allergy symptoms, contact with the fish according to work tasks and *Trisakis*-170 results.

allergy groups	fish-processing work tasks*						
	A1 (3)^¶^	A2	A3 (1)	B1	B2	B3	TOTAL
**BA1**	-	-	-	35 (3)	7	5	47
**BA2**	-	-	-	43 (2)	5	8	56
**BA3**	-	-	-	36	7	4	47
**BA4**	-	-	-	33 (2)	3	6	42
**TOTAL**	268 (3)	39	64 (1)	147 (7)	22	23	563

BA1: cutaneous symptoms; BA2: rhino-conjunctivitis; BA3: respiratory symptoms; BA4: two or

more categories; A1-A3: non-allergic subjects working at line (1), indirect contact with fish (2), or no

contact with fish (3), respectively; B1-B3: allergic subjects working at line (1), indirect contact

with fish (2), or no contact with fish (3), respectively.

*Numbers in parenthesis indicated the number of *Trisakis*-170-positive sera for each group.

^¶^This subgroup includes the borderline serum.

### IgE determinations to Ani s 1 and Ani s 7 allergens

A general screening for the presence of specific anti-*Anisakis* IgE antibodies in the overall sampled population (n = 600) working at marine fish processing facilities was done using the indirect ELISA *Trisakis*-170 kit (Universidad de Santiago de Compostela, Spain). This test uses two recombinant major allergens (Ani s 1 and Ani s 7) as target to assess IgE sensitization to *Anisakis* sp. [9]. Interestingly, this test proved to be adequate for detection of true *Anisakis* infections [19] and was able to detect 100% of gastroallergic anisakiasis and 95% of cases of *Anisakis* sensitization associated with chronic urticaria [20]. Moreover, this method can be considered as a gold standard for specificity as the Ani s 7 recombinant sequence used in the kit has no sequence identity with any known human allergen in line with the FAO/WHO criteria (https://fermi.utmb.edu/). Sensitivity defers between recombinant allergens; Ani s 1 is less sensitive (61%; 95% confidence interval, CI 54–68%) compared to Ani s 7 (93%; 90–98%) [21].

Briefly, wells in columns 1, 4, 7, and 10 of the ELISA plates provided contain rAni s 1, wells in columns 2, 5, 8, and 11 contain a truncated sequence of rAni s 7 and the remaining columns (3, 6, 9 and 12) contain the blocking solution alone (controls). To test the sera, 100 μl of undiluted serum was added to each well and incubated for 30 min at RT with shaking (750 rpm). Then, both the washing steps and the detection of IgE antibodies were done using the reagents provided with the kit and following the kit instructions. SigmaFast OPD (Sigma-Aldrich, Madrid) was used as substrate for peroxidase. Optical densities (ODs) at 492 nm were calculated by subtracting the OD value produced by the same serum in the absence of antigen.

### IgE determinations to a whole *Anisakis* crude extract (CE)

*Anisakis* CE was prepared by Dr. Cuéllar (Departamento de Parasitología, Universidad Complutense, Madrid, Spain) using *A*. *simplex* s. l. (sensu lato) L3 larvae as previously described [21]. Briefly, the wells of Greiner Bio-One ELISA plates (Greiner Bio-One España, S.A.U., Madrid, Spain) were coated with *Anisakis* CE at 5 μg/mL in 0.05 M carbonate-bicarbonate buffer pH 9.6 at 4ºC overnight. Afterwards, the plates containing the antigen were washed three times with TBS-T buffer (50 mM Tris-HCl, pH 7.4; 150 mM NaCl containing 0.05% Tween-20) and blocked with 1% non-fat skimmed milk in TBS (TBS-SM) during 1 h at RT. In parallel, several empty ELISA plates were also blocked with TBS-SM to serve as control. Then, the plates (with or without antigen) were aspirated, the wells filled in duplicate with 100 μL of each serum, previously diluted 1:1 in TBS-SM containing 0.05% Tween-20, and incubated for 30 min at RT with agitation at 750 rpm. Finally, the plates were washed three times with TBS-T and the IgE anti-CE revealed using the same secondary reagents and procedures included with the *Trisakis*-170 kit. Mean OD values for each serum were obtained subtracting the OD values obtained with the corresponding wells without antigen from the wells containing antigen.

### IgE determinations by ImmunoCAP

In addition to *Trisakis*-170 and *Anisakis* CE ELISA determinations, a subsample of sera (n = 76) was also tested for the presence of anti-*Anisakis* IgE antibodies using a commercial test (ImmunoCAP, ThermoFisher Scientific). ImmunoCAP determinations were done automatically at the Laboratory of Immunology (Complejo Hospitalario Universitario de Santiago-CHUS, Santiago de Compostela, Spain). Chosen serum samples for ImmunoCAP determinations included all sera tested positive by *Trisakis*-170 (n = 11, see Results section) and randomized sera from subgroups BA1 (n = 20), subgroup BA2 (n = 18), subgroup BA3 (n = 12) and subgroup BA4 (n = 15). ImmunoCAP test shows high sensitivity (98%; 95% CI 97–100%), but poor specificity (78%; 95% CI 73–83%) [22].

### IgG determinations to *Trichinella* antigens

In order to explore sensitization to another food-borne helminth endemic in some Croatian areas, we tested anti-*Anisakis* seropositive and seronegative sera for the presence of IgG antibodies to *Trichinella* spp. A standard protocol of indirect ELISA using *Trichinella* excretory and secretory antigens (ESA) was employed and results were confirmed by Western Blotting (WB) [23].

### Human leukocyte allergen (HLA) genotyping of susceptibility to *Anisakis*

In total 208 sera; 159 (27%) of fish processing workers and 49 (11%) of controls; were HLA typed, including all 11 anti-*Anisakis* IgE seropositive or suspicious participants. Genomic DNA was extracted from EDTA-blood samples using High Pure PCR Template Preparation Kit (Roche Diagnostics GmbH, Germany). HLA-DRB1, HLA-DQA1 and HLA-DQB1 alleles were detected by PCR-sequence specific oligonucleotide probing method, using commercially available Immucor Lifecodes HLA-SSO typing kit (Immucor Transplant Diagnostics, Inc, Stamford, USA) and standard polymerase chain reaction sequence specific priming protocol for Olerup SSP typing kits (Olerup GmbH, Vienna, Austria).

HLA-allele and genotype frequencies were calculated by direct counting. Departure from Hardy-Weinberg equilibrium (HWE) was assessed by standard frequencies tests: chi-square, exact, and permutation tests; within the R software version 3.5 (http://www.r-project.org/). The loci HLA-DQA1*01:02, HLA-DQA1*05:05 and HLA-DQB1*03:01 deviated from HWE and were excluded from analysis. The final set included 55 HLA-alleles: 25 (46%) common, 20 (36%) low frequency and 10 (18%) rare alleles.

Haplotypes were estimated based on HLA-DRB1, HLA-DQA1, and HLA-DQB1 4-digit typing data using Expectation-Maximization algorithm within the Arlequin v3.5.2.2 software (http://cmpg.unibe.ch/software/arlequin35).

### Statistical calculations

When adequate, statistic comparisons between prevalences of anti-*Anisakis* IgE antibodies obtained either by *Anisakis* CE ELISA, *Trisakis*-170 ELISA or by ImmunoCAP (dependent variable) and its Fisher’s confidence intervals were calculated by WinPepi 11.65 (http://www.brixtonhealth.com/pepi4windows.html). Significance and strength of potential associations between the dependent, and independent variables (gender, age, occupational contact and safety, behavioral risk factors, diet, health status) were inferred with univariate logistic regression analysis using the function logistf (logistf package, the R software version 3.5). The method employs Bayesian approach to fit a logistic regression model using Firth's bias reduction method, standardly used to reduce bias due to low event rate in odds ratio estimates [24].

Odds ratios (OR) were considered statistically significant if their 95% confidence interval (CI) did not include 1. The statistical significance was further corroborated by p-values obtained from regression model’s log-likelihood-ratio statistics, and Fisher exact test. The usage of Firth's method and a sufficient number of events per parameter in univariate logistic regression model assured reliable estimates of regression coefficients and statistical significance [25].

As genetic mechanisms underlying potential associations between HLA-alleles and seropositivity are not known, we tested these associations using allelic, dominant, and recessive models while applying statistical methods described above. The additive model was not built as it includes more than two genotype categories, which increases the number of parameters in a logistic model affecting the reliability of regression coefficient estimates. Haplotype associations with seropositivity were tested within the Arlequin v3.5.2.2 software.

Given that anti-*Anisakis* seropositivity is a rare event in sampled fish-processing workers and that large sample size replication studies on this population are not likely, we applied exploratory association analysis using a significance level of 0.1.

Because not all Ani s 1 and Ani s 7-sensitized workers showed to be positive to the whole-nemetode extract, statistical calculations were performed only for sera testing positive with the *Trisakis*-170 kit.

## Results

The mean ± SD age of fish processing workers, of whom the majority were women (73%, p<0.001), was 46 ± 10 years. Women also predominated in controls (63%, p<0.001) who were, overall, younger than the processing workers (39 ± 11 years, mean difference of 6 years, 95% CI 5.0–7.6). The median age of the seropositive fish processing workers, of whom five (45%) were women, was 56 years (range 30–63) (**S2 Fig**).

### IgE response to Ani s 1 and Ani s 7 allergens

Including the borderline serum testing positive in a first run and borderline in a second run with the *Trisakis*-170 kit (**Tables 1** and **2**), 11 out of 600 sera from fish processing workers tested positive for Ani s 7 IgE antibodies (prevalence 1.8%, Fisher’s exact 95% CI 0.9–3.3%), of which four were both Ani s 1 and Ani s 7 positive (0.7%, Fisher’s exact 95% CI 0.2–1.7%).

**Table 2:** Selected attributes of *Anisakis* spp. seropositive individuals working in Croatian fish processing industry.

serum #	birth year	gender	center & facility	job title	free-time fishing	raw/salted/marinated fish consumption	frequency of fish consumption	preparation of dish	type of consumed fish	acute allergy symptoms	chronic diseses	Ani s 1	Ani s 7	ImmunoCAP	*Trichinella* ELISA	*Trichinella* WB
52	1959	M	1A	fish seller	+	+	few x/ week	B, G, M	SPF, FF	U, Rh	CV	**1.888**	**0.934**	**9.75**	- (4.9%)	-
111	1966	F	1A	fish cleaning and salting	-	+	1x/ week	B, G, R	SPF	U	RA	0.006	**0.124**	**0.54**	-	-
128	1969	F	1A	fish sorting	-	+	every day	B	SPF, FF	C	CA	**0.135**	**0.352**	**17.0**	**+ (14.3%)**	-
216	1959	M	2C	owner	+	+	1x/ week	G, M	FF	C, Rh	-	0.004	**0.503**	**4.99**	**+ (18.6%)**	-
244	1979	M	1A	fish processing	+	+	1x/ week	G	SPF	-	CV	-0.003	**0.161**	**1.94**	**+ (16.6%)**	-
310^a^	1954	M	3E	can sterilisation	+	+	few x/ week	B, G	SPF	-	-	0.004 (0)	**0.06** (0.042)	0.24	**+ (23.9%)**	-
321	1971	F	3E	packaging	+	+	few x/ week	B	SPF	-	P	**0.873**	**1.212**	**3.66**	-	-
446	1957	M	3H	load of raw fish	+	-	1x/ week	G	FW	C, Rh, DC	CA	-0.003	**0.105**	0.23	-	-
453	1957	M	3H	unloading of raw fish	-	-	1x/ week	B, G	SPF	U	-	-0.006	**0.198**	**0.89**	-	-
525	1961	F	4J	commerce	-	-	1x/ week	B, G	SPF, FF	-	-	-0.002	**0.213**	<0.1	-	-
595	1987	F	4J	fish sorting	-	-	few x/ week	G	SPF	U	D, T	**0.132**	**0.173**	<0.1	**+ (17.8%)**	-

Center 1 Brac: A Sardina facility; Center 2 Sinj: B Conex, C Felicita, D Trenton; Center 3 Zadar: E Sali Mardesic, F Omega Benkovac, G Mislov, H Ostrea, I Noclerius; Center 4 Rovinj: J Mirna; F: female; M: male; B: boiled fish; G: grilled fish; M: marinated fish; R: raw fish; SPF: small pelagic fish; FF: finfish; FW: freshwater fish; U: urticaria; Ri: rhinitis; C: conjunctivitis; DC: dry cough; CV: cardiovascular diseases; CA: chronic allergy; P: psychosis; D: diabetes, T: thyroidal imbalance; RA: rheumatoid arthritis. The values in Ani s 1 and Ani s 7 columns show ODs obtained in ELISA for each serum (positives in bold). Cut-off values were OD = 0.09 for Ani s 1 and OD = 0.05 for Ani s 7. The values in ImmunoCAP column show antibodies kU/L obtained using the whole-nematode extract by commercial ImmunoCAP for each serum (positives in bold). Cut-off value is 0.35 kU/L. Cut-off values (shown in parenthesis) for *Trichinella* ELISA was 11.8%. In bold: seropositive. ^a^borderline serum. In parenthesis are Ani s 1 and Ani s 7 OD values of the second testing.

Ten of 11 positive sera were from workers reporting tasks that necessitate a direct contact with fish (subgroups B1 = 7 sera, and A1 = 3 sera). However, these differences did not reach statistical significance (OR = 4.5; 95% CI 0.6–35.1, p = 0.186). Interestingly, most of positive sera (7/11) were also from workers reporting allergies in the questionnaire: cutaneous symptoms (subgroup BA1 = 3 sera), rhino-conjunctivitis (subgroup BA2 = 2 sera) or combined symptoms (subgroup BA4 = 2 sera). Comparing the proportions of seropositive sera in the population of allergic versus non-allergic workers we obtained a significant relationship when we excluded the borderline serum from the analysis (OR = 4.6; 95% CI 1.2–18.2, p = 0.036). In case that the said serum was included, the association was significant at the 0.1 level (OR = 3.5; 95% CI 1.0–12.0, p = 0.052).

In contrast to the population of fish processing workers, all 446 control sera tested anti-*Anisakis* seronegative with Ani s 1 and Ani s 7 (0%, Fisher’s exact 95% CI 0–0.8%) demonstrating significantly lower prevalence than in fish processing workers (Fisher’s exact p = 0.006).

### IgE response to *Anisakis* CE antigens

The IgE response to *Anisakis* CE antigens in fishing processing workers with completed questionnaires (n = 563; 37 excluded; see Table 1), was positive in 21 tested sera. Among them, 10 sera tested positive in the group B (subjects reporting allergies in the questionnaire; n = 192; 5.2%) and 11 sera were positive in group A (subjects not reporting allergic symptoms; n = 371; 3%). No significant differences (p = 0.240; Fisher’s exact test) were obtained between both groups. Also, was observed that all positive sera to CE antigens in group B corresponded to individuals working in direct contact with fish (subgroup B1; n = 146, 6.9%), while in the group A (not reporting allergies in the questionnaire) 9 subjects worked in direct contact with fish (subgroup A1; n = 268, 3.3%) and 2 subjects worked in other tasks (subgroup A3; n = 64, 3.1%). Again, no statistical differences were observed between these subgroups (p = 0.140; Fisher’s exact test). Regarding the positive sera in subgroup B1 (n = 10), 4 sera were from subjects reporting cutaneous allergic symptoms (subgroup BA1; n = 47), 3 subjects reported rhinoconjuctivitis (subgroup BA2; n = 56), 1 subject indicated respiratory symptoms (subgroup BA3; n = 47) and 2 subjects reported symptoms from several categories (subgroup BA4; n = 42). On the other hand, excluding the sera testing positive to Ani s 7 (i.e., those suffering a previous infections by *Anisakis* spp.) only 5/192 (2.6%, 95% CI 0.9–6.0%) sera tested positive to CE in the allergic subpopulation (subgroups BA1 = 2; BA2 = 1; BA3 = 1 and BA4 = 1). These figures were similar to those obtained for the non-allergic subpopulation for which 7/371 (1.9%, 95% CI 0.8–3.9) were positive to CE antigens (subgroups A1 = 4; A2 = 1 and A3 = 1).

As all positive sera to *Anisakis* CE in group B (n = 10) and 9/11 positive sera in group A fell within subgroups B1 and A1, respectively (both corresponding to workers in direct contact with fish), we analyzed whether seroprevalences in subjects working in direct contact with fish (either reporting allergic or non-allergic symptoms; subgroups B1 and A1, respectively) have a significantly higher number of sensitizations to *Anisakis* CE than the remaining workers (subgroups B2 + B3 + A2 + A3; n = 149). Again, the Fisher’s exact test revealed no statistically significant association (OR = 0.31; 95% CI 0.072–1.37, p = 0.120).

### Anti-*Anisakis* IgE response measured by ImmunoCAP

In total seven out of 11 *Trisakis*-170 positive sera also tested positive to ImmunoCAP (sensitivity 63%, 95% CI 31–88%), whereas the results of 65 *Trisakis*-170 negative sera were fully concordant with ImmunoCAP (specificity 100%, 95–100%). By categories (see **Table 1**), the ImmunoCAP positive sera were from subgroups BA1/B1 (2 sera), BA2/B1 (2 sera), BA4/B1 (1 serum), and A1 (2 sera). ImmunoCAP values (kU/L) are included in **Table 2**.

### Anti-*Trichinella* IgG reactivity

Individual anti-*Anisakis* positive sera by *Trisakis*-170, including the borderline negative serum (n = 11), and a random sample of anti-*Anisakis* negative sera (n = 11) were also tested for IgG antibodies to *Trichinella* spp. While the ELISA method detected eight anti-*Trichinella* positive sera, WB was negative for all of them (Table 2).

### Risk factors in fish processing workers

The results of univariate analyses of associations between anti-*Anisakis* seropositivity and sociodemographic, behavioral, or occupational safety attributes of workers are presented in **Table 3**.

**Table 3:** Sociodemographic, behavioral, and occupational safety attributes of Croatian fish processing workers (n = 600) associated with the anti-*Anisakis* seropositivity at the significance level of 0.1.

Attribute		OR	95% CI for OR		p-value^a^	
**Sociodemographic**	**Male gender**	**3.4**	**1.1**	**11.1**	**0.042**	
	**Older age [years]**	**1.1**	**1.0**	**1.2**	**0.091**	
**Occupational safety**	**Wears protective gloves**	0.6	0.1	5.6	0.581	
	**face mask**	**0.2**	**0.001**	**1.3**	**0.094†**	
	**goggles**	0.7	0.01	5.3	0.762	
	**Direct contact with fish**	2.6	0.6	24.4	0.152	
**Behavior**	**Fishes in free time**	**11.4**	**3.5**	**38.7**	**< 0.001**	
	**Fish processing technique used at home to prepare fish**					
	Marinates	**6.7**	**1.2**	**25.7**	**0.031**	
	Prepares raw fish	**9.0**	**0.9**	**46.0**	**0.059†**	
	Salts	NA				
	Smokes	NA				
	Cooks	1.8	0.6	6.4	0.317	
	Grills	0.4	0.1	2.1	0.235	
	**Fish prepared and consumed at**					
	Home	1.0	0.1	>50	0.974	
	Restaurant	4.8	0.5	22.7	0.144	
	Supermarket	0.7	0.01	5.6	0.977	
	**Diet**					
	Eats meat	**0.1**	**0.01**	**0.6**	**0.023**	
	Consumes fish less than once weekly	**0.1**	**0.001**	**0.8**	**0.021**	
	Consumes fish (YES/NO)	0.6	0.1	82.3	0.768	
	Consumes thermally unprocessed fish	2.3	0.7	8.1	0.162	
	**Type of fish consumed:**					
	marine finfish	1.1	0.3	3.6	0.835	
	small marine pelagic fish	0.6	0.2	3.2	0.508	
	freshwater	2.4	0.3	10.7	0.376	
	**Smoking**					
	Non-smokers vs smokers	**0.6**	**0.2**	**2.4**	**p = 0.51**	Overall, for all categories
	Ex-smokers vs smokers	**4.5**	**1.0**	**17.6**	**p = 0.05**	p = 0.060

See the Methods section for the rationale of the significance level

^a^Shown is the p-value of logistic regression model’s likelihood ratio test. All findings are additionally supported with the Fisher’s exact p-value < 0.1, except for the p-values marked with † for which Fisher exact test reaches a p-value between 0.1 and 0.2.

The strongest risk factor for acquiring anti-*Anisakis* seropositivity is *fishing in the free time*, followed by the *traditional fish processing* techniques used *at home* (**Table 3**). Contrary, no association was observed for *direct contact with fish on a production line* in a factory, *wearing gloves* or *protective goggles*. It should be stressed, however, that the majority of workers (92%) wore protective gloves even if not in direct contact with fish, minimizing the risk for *Anisakis* sensitization via skin contact.

Except for the *grilling* and the *cooking*, other fish processing techniques were rarely used by participants at home (**S2 Table**). Nevertheless, *marinating or raw fish preparation* showed strong association with the seropositivity (**Table 3**). No seropositive workers *salted or smoked fish* as it requires particular equipment, therefore the estimation of their risk was unfeasible.

Dietary habits showed that *meat consumption* and *fish consumption less than once weekly* were strong protective factors against *Anisakis* sensitization (**Table 3**). Contrary, *fish consumption* per se or *consumption of thermally processed fish* weren’t identified as significant factors. Interestingly, the *type of fish* or the *place where fish is prepared and consumed* were not associated with seropositivity, even though more than 63% participants declared consumption of the *marine small oily (pelagic) fish*, which traditionally is the only fish type prepared without gutting.

*Male gender* and *older age* were risk factors; men were 3.4 times more likely to become seropositive than women, and *older age* increased this risk by 10% annually (**Table 3**). A significant interaction between *sex* and *age* was shown as seropositive men were much older than seropositive women (Mann-Whitney test, p = 0.002, **S2 Fig**). Given the fact that men are more inclined towards fishing in spare time, after removal of those who *fish in their free time*, there were 1% of seropositive participants both in men (1 out of 111) and women (4 out of 391). However, as the observation is derived from just five events in total of 502 participants which is less than minimum 10, no formal testing was conducted.

In total 563 fish processing workers answered the question on *chronic allergies*; of whom 32 (6%) self-reported chronic allergies. The *chronic allergy* issue was explained to workers as the presence of any type of allergy symptoms they had before their employment (i.e. childhood and teenage), and *acute allergy* as an entity they have acquired since being employed in fish processing facility. Seronegative workers self-reported chronic allergies less frequently than seropositive workers (6% vs 27%; OR 0.2, 95% CI 0.04–0.6) or the controls (6% vs 14%; OR 0.4, 95% CI 0.2–0.6). When we compared the prevalence of exclusively acute allergic symptoms in workers with that of allergy symptoms in controls we found that urticaria and rhinitis were frequent in seropositive workers, less so in seronegative workers, and were present in just 2% of healthy controls (**Table 4** and **S3 Fig**).

**Table 4:** Prevalence of acute allergy related symptoms in seropositive and seronegative fish processing workers, and of allergy symptoms in healthy controls in Croatia.

	n (%)			OR (95% CI)^d^		
Acute allergy symptoms	Seropositive, n = 8^a^	Seronegative, n = 501^b^	Healthy control, n = 443^c^	Seropositive vs Seronegative	Seropositive vs Healthy	Seronegative vs Healthy
Urticaria	3 (38%)	56 (11%)	7 (2%)	5.02 (1.14, 19.37)**	37.0 (7.5, >100)**	7.38 (3.62, 17.39)**
Conjunctivitis	1 (13%)	56 (11%)	6 (1%)	1.58 (0.16, 7.41)	13.5 (1.3, 77.3)**	8.54 (4.03, 21.47)**
Rhinitis	2 (25%)	36 (7%)	11 (2%)	4.91 (0.88, 20.01)*	14.5 (2.5, 64.6)**	2.95 (1.55, 6.05)**
Non-productive cough	1 (14%)	55 (11%)	4 (1%)	0.47 (0.00, 3.89)	5.7 (0.0, 61.1)	12.14 (5.13, 37.35)**
Bronchitis	0 (0%)	7 (1%)	0 (0%)	3.88 (0.03, 36.74)	Zero prevalence both	13.45 (1.63, >100)**

^a^includes the borderline case and excludes data on three workers reporting chronic allergies

^b^excluding data on 29 workers with chronic allergies and 59 missing data

^c^3 answers were missing

^d^odds ratio calculated with Firth's bias reduction method; Significant at: ** - 0.05 level, * - 0.01 level

### Human leukocyte allergen (HLA) and *Anisakis* sensitization

To investigate if the *Anisakis*-induced allergic response might be mediated by HLA gene polymorphisms, we assessed the association between seropositivity and HLA alleles coding for different protein types within DRB1, DQA1, and DQB1 loci. We observed a high level of HLA allelic diversity (homozygotes prevalence 0–4%). The majority of alleles were not associated with the seropositivity under any genetic model tested (logistic regression, p≥0.110). However, six HLA alleles within the DRB1 gene increased the risk of seropositivity under: dominant, allelic and/or recessive models (Table 5). All of these associations were identified as strong (OR≥3.5) and were additionally supported by the results of Fisher exact tests, which were significant at the 0.1 level (Table 5), with the precision of estimates being somewhat lower. For other genes, we found similar, but fewer significant associations with only one allele associated with increased risk for acquiring seropositivity in the DQA1 gene, and three alleles identified in the DQB1 gene. Of the latter, only the allele HLA-DQB1*05:02 was allied with no IgE hypersensitivity.

**Table 5:** Associations to anti-*Anisakis* seropositivity in Croatia among 10 HLA alleles from 11 seropositive (including the borderline case), 148 seronegative fish processing workers and 49 controls, at the significance level of 0.1.

	Allele frequency		Allelic model			Dominant model			Recessive model		
HLA allele	cases	controls	p-value^a^	OR	95% CI	p-value	OR	95% CI	p- value	OR	95% CI
HLA-DRB1*11:01	9.1%	9.4%							0.082†	7.94	0.72- 53.86
HLA-DRB1*11:04	13.6%	4.8%	0.076	3.46	0.86- 10.70	0.045	4.53	1.04- 16.43			
HLA-DRB1*13:02	27.3%	8.6%	0.057	3.87	0.96- 12.14	0.053	4.25	0.98- 15.31			
HLA-DRB1*3:01	9.1%	6.3%							0.082†	7.94	0.72- 53.86
HLA-DRB1*4:04	18.2%	3.0%	0.029	7.29	1.28- 30.72	0.028	7.75	1.29- 35.76			
HLA-DRB1*7:01	18.2%	8.9%							0.010	56.43	2.84- >100
HLA-DQA1:2:01	18.2%	7.9%	0.090	2.81	0.83- 7.79						
HLA-DQB1:2:02	18.2%	7.9%	0.090	2.81	0.83- 7.79						
HLA-DQB1:5:02	0.0%	11.4%	0.098†	0.17	0.00- 1.27						
HLA-DQB1:6:05	9.1%	0.0%	0.010	55.05	2.85- >100	0.010	56.43	2.84- >100			

See the Methods section for the rationale of the significance level

^a^Shown is the p-value of logistic regression model’s likelihood ratio test. All findings are additionally supported with the Fisher’s exact p-value < 0.1, except for the p-values marked with † for which Fisher exact test reaches a p-value between 0.1 and 0.2.

In respect to the haplotype association, we demonstrated significant associations of anti-*Anisakis* seropositivity with five haplotypes, containing majority of HLA alleles listed in **Table 6**. The exception were HLA-DRB1*03:01, HLA-DRB1*11:01, and HLA-DQB1*05:02 loci.

**Table 6:** Associations to anti-*Anisakis* seropositivity in Croatia among HLA haplotypes from 11 seropositive^a^, 148 seronegative fish processing workers and 49 controls.

Allelic model				
HLA haplotype DRB1 DQA1 DQB1	Haplotype frequency		p-value^b^	p-value Fisher
	cases	controls		
**7:01 2:01 2:02**	18.2%	6.6%	1.09E-14	0.064
**11:04 5:05 3:01**	13.6%	3.6%	3.33E-16	0.054
**4:04 3:01 3:02**	9.1%	0.8%	1.11E-16	0.024
**13:02 1:02 6:05**	4.5%	0.0%	0	0.053
**13:02 3:01 6:04**	4.5%	0.0%	0	0.053

^a^including the borderline case

^b^Shown is the p-value of logistic regression model’s likelihood ratio test. All findings are additionally supported with the Fisher’s exact p-value < 0.1

## Discussion

This is the first study systematically evaluating the risk of *Anisakis* allergen sensitization in a representative sample of 600 employees of fish processing facilities and 466 working-age blood donor controls, using highly specific and sensitive serodiagnostic test. Also tested are associations of *Anisakis*-sensitization in fish processing workers to a number of sociodemographic, behavioral, health-related, genetic, and professional attributes. Given that the knowledge on risk factors for *Anisakis* sensitization is incomplete, often contradictory and vague, and since the replication of the study is unlikely as it would have required repeated efforts to achieve formal cooperation of entire industry, we performed exploratory association analysis. Exploratory nature together with a low number of observed events might have affected the precision of some estimates and conditioned the emergence of some false-positive interactions. Nevertheless, association results presented here are mainly corroborated with the strength of associations, their consistency, biological plausibility, and/or coherence with previous knowledge, as described in detail below. More importantly, we argue that the study exhaustively covered almost all marine fish processing workers in Croatia, precisely reflecting real-time *Anisakis* sensitization status within the industry. This sets a baseline for the future tracking of sensitization in the sector already under the influence of wide array of allergens.

### Risk factors for *Anisakis* sensitization in fish processing workers

Higher anti-*Anisakis* seroprevalences in workers (1.8%, 95% CI 0.9–3.3%) compared to controls (0%, 0–0.8%) suggests an increased risk of acquiring *Anisakis* seropositivity in fish processing industry. This was also postulated in France, Italy, South Africa and Spain [14,15,26–29], but the body of evidence was limited to case reports, studies with small sample size and/or non-representative sample or cross-sectional studies without control groups of individuals outside of the fish processing industry. However, in our study the highest risk for *Anisakis* sensitization among the workers was fishing in the free time (OR 11.4), suggesting that the professional exposure in studied environment does not represent a major risk factor for fish-processing workers when appropriate safety measures such as gloves, masks and goggles are in place. Rather, the association between IgE sensitization and fishing in free time, which is found in this subpopulation and accompanied with 5 times higher frequency of eating raw fish in amateur fisherman than in those who do not, implicate this behaviour as risky (Table 2). The fact that all seropositive workers tested by *Trisakis*-170 were positive to Ani s 7, which reveals true *Anisakis* infections [19], confirms that anti-*Anisakis* IgE antibodies in our study were induced by an unsuspected previous *Anisakis* infection rather than to an environmental exposition of workers to *Anisakis* allergens. Although fish workers may be more exposed to *Anisakis* aeroallergens than normal population, this seems not to be relevant to sensitize against major Ani s 1 and Ani s 7 secretory allergens.

While eating fish per se (OR 0.6, 95% CI 0.1–82.3, p = 0.768) or eating thermally unprocessed fish (OR 2.3, 95% CI 0.7–8.1, p = 0.162) were not risk factors in our study, eating meat and the frequency of fish consumption were. The reason for this contradiction may be that workers who didn’t eat meat actually ate fish more frequently (no vegetarians or vegans were observed, reflecting dietary habits of a typical Croatian).

An alternative explanation for the lack of association between *Anisakis* IgE sensitization and exposure to *Anisakis* allergens in the population of fish processing workers is that Ani s 1 and Ani s 7 allergen present in the *Trisakis*-170 kit are not adequate to measure sensitization by cutaneous and/or respiratory routes. To cover this possibility, the sera of the entire population working in the fish industry, and reporting allergy status in the questionnaire (n = 563), were tested for IgE antibodies to *Anisakis* CE antigen in indirect ELISA. In this case, an increased number of fish-processing employees has been found positive to *Anisakis* CE (3.9%, 2.4–6.0%) in comparison with *Trisakis*-170 (1.8%, 95% CI 0.9–3.3%). At a first glance, such difference may suggest: i) that the working population might be sensitized to other relevant *Anisakis* allergens present in CE and not present in the *Trisakis*-170 kit (Ani s 1 and Ani s 7, only), or alternatively, ii) that some allergens present in the *Anisakis* CE cross-reacted with other allergens to which some workers are sensitized. To distinguish between these two possibilities, the frequency of IgE sensitization to *Anisakis* CE was compared within the subpopulations of workers reporting versus those non-reporting allergic symptoms, as well as between subjects working in direct contact with fish with respect to the remaining subjects working in other tasks. We found no significant differences in the proportion of *Anisakis* IgE sensitization for any of the above comparisons, which suggests that the greater amount of positive patients to *Anisakis* CE, compared to *Trisakis*-170, is more probably due to cross-reactivity with other allergens than to sensitizations to *Anisakis* by skin and/or respiratory routes. Similarly, but with the lower level of evidence, Mazzucco et al. [14] showed that seroprevalence among fishermen/ sailors compared to fish industry workers was 6.7-fold greater, whereas the skin contact frequency was same among two groups. Authors unexpectedly found an inverse association between dietary fish intake and seroprevalence, and interpreted the anomaly by potentially reduced fish consumption in workers suffering allergy. Nevertheless, since a great majority of subjects in that study [14] worked in direct contact with fish (417 versus 148) a bias could be introduced and this conclusion should be taken with some caution.

Another reason for dominant behavioral instead of occupational risk factors could be that this subpopulation shows a more frequent habit of eating raw small pelagic fish, which unlike the processed anchovy and sardine, does not undergo veterinary inspection. The protective clothing (goggles, gloves, mask), being a prerequisite for the application of HACCP (Hazard Analysis of Critical Control Points) principles in Croatian fish processing industry [30], could also play a role. Workers wearing a face mask seemed to be protected (p<0.1), but because all *Anisakis*-sensitized workers wore gloves and only 20% of all workers wore masks, we were unable to precisely determine the risk of not wearing protective clothing in *Anisakis* sensitization. The effect of protective clothing and occupational allergies in general is not fully resolved. It can be insignificant in adults with atopic dermatitis [31] or represent an occupational allergen per se (natural rubber latex, [32]). Therefore, the odds of protection clothing in *Anisakis* allergy should be carefully considered in future in a differently designed study.

Unlike for other acute allergic symptoms in workers, the occurrence of urticaria and rhinitis (**Table 4**) was significantly higher in seropositive than in seronegative individuals (ORs from 4.9 to 5.0) or controls (ORs from 14.5 to 37.0). This is not surprising because urticaria is typically associated to helminths and blood-sucking arthropods with a cutaneous phase triggering Th2-type response [33]. We also found implication of other allergens in our study by differentiating between chronic and acute allergy, as seronegative workers reported more frequently all of the listed acute allergic symptoms than the controls. Similar to our finding, Jeebhay et al. [34] found that asthma and ocular-nasal symptoms are frequent in marine fish processing facilities. The implication of multiple-allergens sensitization and clinical manifestation of the allergy in case of *Anisakis* is still unresolved. Daschner et al. [35] observed that *Anisakis-*sensitized patients suffering chronic urticaria had also high prevalence of pollen, mold and dander sensitization. House dust mites sensitization was less prevalent in those patients, whom then exhibited allergic rhino-conjunctivitis and bronchial asthma, even in a region with a low burden of mites allergens.

No difference in *Anisakis* sensitization was observed between smoking and non-smoking workers, but ex-smokers were at significantly higher risk of sensitization than smokers, probably due to changes in the nasal mucociliary epithelium typical for ex-smokers. Namely, while the expelling of cigarette irritants through mucosal ciliature in smokers is inefficient, cigarettes induce coughing that enables the clearing process. In ex-smokers, it can take a year for the cilia to recover after cessation, during which ex-smokers can be susceptible to chest infections [36]. Whether the same mechanism has a role in *Anisakis* sensitization still remains to be tested.

Older male workers were at higher risk to *Anisakis* sensitization, contrasting a retrospective survey in French hospitals on anisakiasis incidence which demonstrated female dominance and younger average age of cases [37]. However, the latter encompassed broader range of species (genera *Pseudoterranova* and *Anisakis*) and surveyed different disease types (“*esophageal*, *gastroduodenal*, *allergic*, *eosinophilic granuloma or another form*”) in contrast to our study that focuses on sensitization. The predominance of *Anisakis*-sensitized women was previously found in Japan and South Korea [38,39] related to their increased consumption of raw fish, but not in USA or Croatia [9,40]. This suggests that age and gender susceptibility are strongly influenced by country-specific diversity of sociodemographic, behavioral and occupational habits of the population under study.

### Seroprevalence of anti-*Anisakis* IgE and anti-*Trichinella* IgG

The Ani s 7 allergen [41] was present in all seropositive individuals, which is in agreement with previous studies that recognize it as the most relevant *Anisakis* allergen, including patients with *Anisakis*-induced chronic urticaria [20]. By contrast, Ani s 1 tested positive only in four individuals, but the inclusion of this allergen in the *Trisakis*-170 is of interest since IgE antibodies to Ani s 1 may be present in a few number of cases not detected by Ani s 7 [20]. The measurement of the IgE response to this allergen is also of interest since in the absence of an additional exposure to infection, IgE levels to Anis s 1 seem to persist, or even increase after sustained oral exposition to *Anisakis* allergens present in fishery products [10].

Interestingly, when the sensitization to the nematode whole extract presented in ImmunoCAP has been measured in a sera subsample (n = 76; fish-processing employees that were Ani s 1 and Ani s 7 seropositive or seronegative, but suffered one or more allergy symptoms), positive reaction was observed only in seven *Trisakis*-170-seropositive sera. This is surprising at the first glance since ImmunoCAP may contain *Anisakis* allergens recognized by cross-reactive antibodies [23], therefore giving false positive results. A possible explanation for these results is that Ani s 1 and/or Ani s 7 major allergens might not be well represented in the current pool of antigens used in ImmunoCAP, which may provoke false negative results when the response to these allergens is dominant. The relatively low IgE response observed with ImmunoCAP for some sera with respect to *Trisakis*-170 (e.g., #321 in **Table 2**) is in agreement with this hypothesis. Moreover, the fact that all ImmunoCAP positive sera fell within the group of *Trisakis*-170 positive workers reinforces our conclusion that the fish-processing environment per se is not associated with risk of *Anisakis*-sensitization. Otherwise, it would be expected that a significant number of workers in contact with *Anisakis* antigens (e.g. through ingestion of processed fish or aerosols in the working environment), tested positive by ImmunoCAP and negative by *Trisakis*-170.

In our previous study [9] we observed that 60% (6/10 cases) of *Trisakis*-170-positive sera were also positive to *Ascaris* sp., *Toxocara* sp. or both (tested by a commercial ELISA for detection of IgG, encompassing a pool of antigens with 95% specificity), suggesting that the output was merely a cross-reactivity, rather than the real status of seroprevalence. This time we tested *Trichinella*-sensitization for two reasons; firstly, because of the historical incidence of acute trichinellosis in the coastal area, although with low incidence (1.7–4.8/ 100,000 inhabitants in Croatia [42]); and secondly, because the migratory larvae of *Trichinella* spp. resembles the human infection model by L3 *Anisakis*. As *Anisakis* seropositive sera tested negative for anti-*Trichinella* IgG, no association was investigated. Cross-reactivity is a major problem in the serological diagnosis of parasitic infections (especially those caused by nematodes), particularly when crude parasite extracts are used, and to a lesser extent the excretory/secretory antigens (ESA). Such cross-reactivity has previously been described among *A*. *simplex* and other nematodes [43,44], and among *Trichinella* spp. and other zoonotic parasites [45,46]. However, studies demonstrated that WBs based on homologous ESA could distinguish between Anisakidae and *Trichinella* seropositivity [47] and that a WB based on *T*. *spiralis* ESA allows the definition of a unique diagnostic pattern for *Trichinella*-infected humans [24].

Difference in seroprevalence of healthy population estimated in Mladineo et al. [9] (2%) and in this study (0%) might have arisen from different structure of two groups. While the former recruited persons ongoing professional and systematic medical examination using stratified sampling, the latter recruited blood donors at sites close to the factories who, by definition, are frequently scrutinized for health and better reflect working population in terms of age. Compared to the sample from 2014 with the mean age of 58 years, participants from this sample were on average 19 years younger (95% CI 17–21).

### HLA and *Anisakis* sensitization

HLA class I and class II proteins play a pivotal role in the adaptive human immune system, the latter mediating specific immunization to the antigen. In this study we identified several HLA class II loci strongly associated with *Anisakis* seropositivity, majority increasing the risk of acquiring seropositivity. This suggests that identified HLA-alleles might be implicated in functional improvement of MHC class II antigen-presenting role by i.e. sustained antigen presentation, in line with described hypersensitivity to *Anisakis* [20]. Similar to our study, Sánchez-Velasco et al. [48] reported that HLA-DRB1*04:04 increased the risk of *Anisakis* allergy by 4.9 times, while DQB1*02:02 was exclusive for *Anisakis*-allergic patients. Moreover, the epitope-binding motif of HLA molecules coded by HLA DRB1*04:04 has been evidenced at least once in the homologous segments of all the characterized allergens of *A*. *simplex* [49]. In addition to HLA-alleles already detected by Sánchez-Velasco et al. [48], we identified seven other risk HLA-alleles (**Table 5**). While some of these alleles might represent spurious association or be in linkage disequilibrium, several findings support their identification. Specifically, DRB1*11:01 allele is involved in presentation of group 2 allergens of *Dermatophagoides* spp. [50] to which all the *Anisakis* allergic patients previously showed sensitization [51]. This is not surprising as both Ani s 1 and 7 are encompassed within major allergens group, being products of the nematode excretory and secretory activity [5]. DQB1*05:02 that we identified as a strong protective factor against *Anisakis* sensitization is also a protective factor against house dust mite–sensitive allergic rhinitis [52]. The list of HLA-alleles and haplotypes we found important for *Anisakis* sensitization, associated with other disorders, is presented in **S3 Table**. It suggests that HLA association with *Anisakis* sensitization is primarily the result of general susceptibility of the seropositive individual to the impairment of immune system, including allergies to major allergens, rather than the nematode per se.

## Conclusion

This is the largest seroepidemiological study that evaluated the risk of *Anisakis* sp. allergens exposure in employees of fish processing facilities, testing the relation of a number of sociodemographic, behavioural, health-related and professional traits of the scrutinised group, and the nematode seroprevalence. While we evidenced significantly higher seroprevalence in fish processing employees compared to the healthy control population, the major risk for the former group turned to be fishing in the free time. The main limitation of our study is the low number of seropositive individuals within fish processing employees, and the disequilibrium between the number of subjects working in direct contact with fish with respect to other workers, but we argue that data-analysis methodology secured reliable estimates and optimal cost/benefit ratio of the research, and that the study exhaustively covered almost all fish processing employees in Croatia, therefore reflecting accurately the contemporary *Anisakis* sensitisation status in the industry.

## Supporting information

S1 Checklist STROBE checklist. (DOCX)

S1 Table Demographic structure of facilities included in the study and attributes of centres encompassing target facilities. (DOCX)

S2 Table Frequency distribution of fish processing techniques that the sample of fish processing Croatian workers employs at home. (DOCX)

S3 Table The list of HLA alleles and haplotypes we found associated with *Anisakis* sensitisation, which have been associated with other disorders. (DOCX)

S1 Data Study design and sampling protocol of *Anisakis* seroprevalence study in fish processing workers. (DOCX)

S2 Data A sample questionnaire distributed to the employees in fish processing industry in Croatia enrolled in this study. (DOCX)

S1 Fig Pie charts showing responses of the employees in Croatian fish processing industry to the distributed questionnaire. (TIFF)

S2 Fig Age distribution according to seropositivity status in Croatia (Positive, Negative) and gender (M-men, W-women). (DOCX)

S3 Fig Prevalence of different acute allergy symptoms in anti-*Anisakis* seropositive and seronegative Croatian workers, and in controls. (PDF)
