# Supplementary material for: Anisakis Sensitization in the Croatian fish processing workers: Behavioral instead of occupational risk factors?
Source: PLoS Negl Trop Dis. 2020 Jan 27;14(1):e0008038. doi: 10.1371/journal.pntd.0008038 (PMC7004557; doi:10.1371/journal.pntd.0008038)
Supplement: S1 Table — (DOCX) [file pntd.0008038.s002.docx]

**S1 Table.** Demographic structure of facilities included in the study and attributes of centres encompassing target facilities.

Distribution of gender in sampled fish processing workers and controls by geographical centers and facilities included in the study. Also shown are the population size and other attributes of geographical centers encompassing target facilities.

| Center | 1 | | 2 | | | | | | 3 | | | | | | | | | | 4 | | Total |
| --- | --- | --- | --- | --- | --- | --- | --- | --- | --- | --- | --- | --- | --- | --- | --- | --- | --- | --- | --- | --- | --- |
| inhabit. | 13,956 | | 24,826 | | | | | | 170,017 | | | | | | | | | | 14,294 | | 223,093 |
|  | A | | B | | C | | D | | E | | F | | G | | H | | I | | J | |  |
|  | F | M | F | M | F | M | F | M | F | M | F | M | F | M | F | M | F | M | F | M |  |
| target sera | 115 | 78 | 61 | 22 | 9 | 2 | 31 | 1 | 33 | 15 | 19 | 4 | 22 | 4 | 81 | 13 | 24 | 3 | 43 | 18 | 600 |
| control sera | 144 | 87 | 52 | 9 |  |  |  |  | 55 | 48 |  |  |  |  |  |  |  |  | 29 | 22 | 446 |
| Center 1 Brac: facility A Sardina; Center 2 Sinj: facility B Conex, C Felicita, D Trenton; Center 3 Zadar: facility E Sali Mardesic, F Omega Benkovac, G Mislov, H Ostrea, I Noclerius; Center 4 Rovinj: facility J Mirna.  Center 1: Brac (43°19′N 16°38′E) is the third largest island in the Adriatic Sea, population 13,956 (1), important sectors are tourism, fisheries, aquaculture and agriculture; Center 2: Sinj (43°42′N 16°38′E) is the center of Cetinska krajina, administratively belonging to Split-Dalmatia County. It has 24,826 inhabitants and important sector include catering trade and a developed business zone (1); Center 3: Zadar (44°6′51''N 15°13′40''E) is situated on the Adriatic Sea as the seat of Zadar County (population 170,017) (1) and the wider northern Dalmatian region. Major industries include tourism, traffic, seaborne trade, agriculture, fishing and fish farming activities; metal manufacturing and mechanical engineering industries; chemicals and non-metal industry; and banking; Center 4: Rovinj (45°04′59''N 13°38′402''E) is a city situated on the north Adriatic Sea with a population of 14,294 (1). Located on the western coast of the Istrian peninsula, it is a popular tourist resort and an active fishing port. | | | | | | | | | | | | | | | | | | | | | |
