# Supplementary material for: Anisakis Sensitization in the Croatian fish processing workers: Behavioral instead of occupational risk factors?
Source: PLoS Negl Trop Dis. 2020 Jan 27;14(1):e0008038. doi: 10.1371/journal.pntd.0008038 (PMC7004557; doi:10.1371/journal.pntd.0008038)
Supplement: S2 Table — (DOCX) [file pntd.0008038.s003.docx]

**S2 Table.** Frequency distribution of fish processing techniques that the sample of fish processing Croatian workers employs at home

|  | **N** | **%^a^** |
| --- | --- | --- |
| Grilling | 501 | 91 |
| Cooking | 267 | 48 |
| Marinating | 22 | 4 |
| Salting | 17 | 3 |
| Raw fish processing | 9 | 2 |
| Smoking | 2 | 0.4 |

^a^Data from 47 questionnaires were missing and were not included in the analysis
