# Supplementary material for: Anisakis Sensitization in the Croatian fish processing workers: Behavioral instead of occupational risk factors?
Source: PLoS Negl Trop Dis. 2020 Jan 27;14(1):e0008038. doi: 10.1371/journal.pntd.0008038 (PMC7004557; doi:10.1371/journal.pntd.0008038)
Supplement: S3 Table — (DOCX) [file pntd.0008038.s004.docx]

**S3 Table.** The list of HLA alleles and haplotypes we found associated with *Anisakis* sensitisation, which have been associated with other disorders.

| **HLA haplotype** | **alleles** | **risk/ disease** | **reference** |
| --- | --- | --- | --- |
| DRB1 DQA1 DQB1 | 07:01 02:01 02:02 | high risk of asparaginase hypersensitivity in acute lymphoblastic leukemia | (4) |
|  | 07:01 02:01 02:03 | high risk of immune dysregulation of Steroid-Sensitive Nephrotic Syndrome | (5) |
|  | 11:04 05:05 03:01 | protective factor of Type I diabetes | (6) |
| DQB1 | 05:02 | reactivation of latent varicella-zoster virus infection | (7) |
|  | 05:02 | disseminated nontuberculous mycobacterial (dNTM) infections | (7) |
|  | 05:02 | clozapine-induced agranulocytosis/ granulocytopenia (CIAG) | (8) |
| DRB1 | 03:01, 07:01 | low serum IgE level production in asthmatics |  |
|  | 11:01 | high serum IgE level production in asthmatics | (9) |
|  | 13:02 | in patients with aspirin-induced urticaria | (10) |
| DQA1 | 02:01 | high serum IgE level production in asthmatics | (9) |
