# Supplementary material for: Anisakis Sensitization in the Croatian fish processing workers: Behavioral instead of occupational risk factors?
Source: PLoS Negl Trop Dis. 2020 Jan 27;14(1):e0008038. doi: 10.1371/journal.pntd.0008038 (PMC7004557; doi:10.1371/journal.pntd.0008038)
Supplement: S1 Data — (DOCX) [file pntd.0008038.s005.docx]

**S1 Data.** Study design and sampling protocol of *Anisakis* seroprevalence study in fish processing workers

The minimal sample size of the study was a priori determined using EpiTools software package (http://epitools.ausvet.com.au) assuming that anti-*Anisakis* seroprevalence in marine fish processing workers is similar to 2%, as estimated previously (2). Given a total of 1149 workers employed in Croatian fish, crustacean and bivalve processing industry in 2016 (3) of which two-thirds (768) were employed in marine fish sector where *Anisakis* sp. exposure is feasible; a minimal sample size of 381 was required in this population of finite size to achieve the marginal error of 1% (confidence interval of 95%). We increased this initial sample size to 600, to ensure sufficient number of seropositive workers for further analyses.

In total 78% of the target population of marine fish processing workers was sampled. Local health providers (Teaching Institute of Public of County of Split-Dalmatia, Zadar and Rijeka, and Department for Transfusion Clinical Hospital Center Split) recruited target subjects that underwent obligatory annual systematic medical examination for working permit. All eligible target participants were adults aged 25 or over who declared healthy, reported the residency in one of four aforementioned geographic areas, and have been working in marine fish processing facility (**S1 Table**). Subjects were excluded if they had acute or chronic infectious disease symptoms at the time of blood sampling. Eligible workers agreed to participate in the study, gave blood and filled anonymous questionnaire (**S3 Data**).

To compare *Anisakis* seroprevalence in fish processing workers in Croatia with that of apparently healthy Croatian adults (controls) employed elsewhere, we recruited participants during volunteering blood donation at sites close to the factories. These participants were also aged 25 or over, and with the residency in one of the four aforementioned geographic areas. A random sampling schema was employed for their recruiting at volunteering blood donation sites. The software SimDis (Monte Carlo simulation; http://www.izor.hr/web/guest/simdis) was used to generate a list of 2,500 random numbers. Independent healthcare workers distributed 2,500 sealed, non-transparent envelopes to these participants at the healthcare provider, of which only a half (1250) contained an invitation to donate blood and the anonymous questionnaire on personal data, and health conditions. Data collection was stopped when the number of healthy controls approached the site-specific number of sampled factory workers. The response rate of controls was 36%, which is almost twice the rate achieved in our previous study (2). The shorter version of the questionnaire was distributed to the control group due to unfeasibility and impracticability, as blood donation campaigns are massive events with limited bed places and high rate of people flow through. The control group questionnaire included personal data (gender, age, contact) and data on health conditions (smoking, presence and type of allergy).

Outcome assessors and data analysts were kept blinded to the distribution of questionnaires to the participants and to group allocation of participants.
