# Supplementary material for: Anisakis Sensitization in the Croatian fish processing workers: Behavioral instead of occupational risk factors?
Source: PLoS Negl Trop Dis. 2020 Jan 27;14(1):e0008038. doi: 10.1371/journal.pntd.0008038 (PMC7004557; doi:10.1371/journal.pntd.0008038)
Supplement: S2 Data — (DOCX) [file pntd.0008038.s006.docx]

**S2 Data**. A sample questionnaire distributed to the employees in fish processing industry in Croatia enrolled in this study.

Number of the sample:___; Facility name:_____________________;

Gender: M F;

Town of residency:_________________; Telephone number:____________.

1. Year of birth:
a) up to 1959
b) 1960 – 1969
c) 1970 – 1979
d) 1980 – 1989
e) 1990 and after

2. Since when are you working in the fish processing sector:
a) 1970 – 1979

b) 1980 – 1989
c) 1990 – 1999

d) 2000 – 2009

e) 2010 – 2015

3. Employment status:

a) Full time

b) Half time

c) Seasonal

4. Do you wear protective equipment at your working place:

a) No

b) Rubber gloves

c) Mask

d) Goggles

5. Do you go fishing in your free time:

a) Yes

b) No

6. Do you salt fish for your own consumption:

a) Yes

b) No

7. Do you eat red meat:

a) Yes

b) No

8. Do you eat fish and fish products:

a) Yes

b) No

9. How often do you eat fish/ fish products:

a) Every day

b) Few times per week

c) Once a week

d) Rarely

10. Do you eat thermally non-processed fish (raw, salted, marinated):

a) Yes

b) No

11. What kind of fish do you eat mostly:

a) Seawater small oily fish (anchovy, mackerel...)

b) Seawater finfish (sea bass, breams, hake...)

c) Freshwater

12. If you eat fish, how do you usually prepare it:

a) Cooked

b) Roasted/ grilled

c) Marinated

d) Salted

e) Smoked

f) Raw (anchovy, sardine, sushi...)

13. Fish dishes that you consume are:

a) Home-made

b) Already prepared from the store

c) Prepared in the restaurant

14. Smoking status:

a) Non-smoker

b) Ex-smoker (I am not smoking more then 1 month)

c) Everyday-smoker

15. Do you have any chronic diseases:
a) Yes

b) No

16. If yes, which one:

a) Lung diseases

b) Cardiovascular diseases

c) Malignant diseases

d) Allergic diseases (skin changes, rhinitis, conjunctivitis, bronchitis, asthma...)

e) Immunity-related diseases (rheumatoid arthritis)

f) Other

17. If you have allergies, which is your common symptom:

a) Urticaria, skin redness (mostly on the hands)

b) Conjunctivitis (redness of the eyes, itchiness, tearing)

c) Rhinitis

d) Coughing, sneezing, shortness of breath

e) Bronchitis, asthma

18. How often do you have allergy symptoms:

a) Frequently

b) Once a month

c) Once in 3 months

d) Once in 6 months

e) Once a year

f) Rarely

g) Never

19. Your symptoms occur more frequently:

In working time

In non-working time (weekends, leave, holidays)

Equally

20. If you are allergic, do you know what you are allergic to:

a) House dust

b) Eggs

c) Fish

d) Ambrosia

e) Peanuts, nuts...

21. Having a family history of allergies:

a) Yes

b) No
