# Supplementary figures and images for: Anisakis Sensitization in the Croatian fish processing workers: Behavioral instead of occupational risk factors?
Source: PLoS Negl Trop Dis. 2020 Jan 27;14(1):e0008038. doi: 10.1371/journal.pntd.0008038 (PMC7004557; doi:10.1371/journal.pntd.0008038)

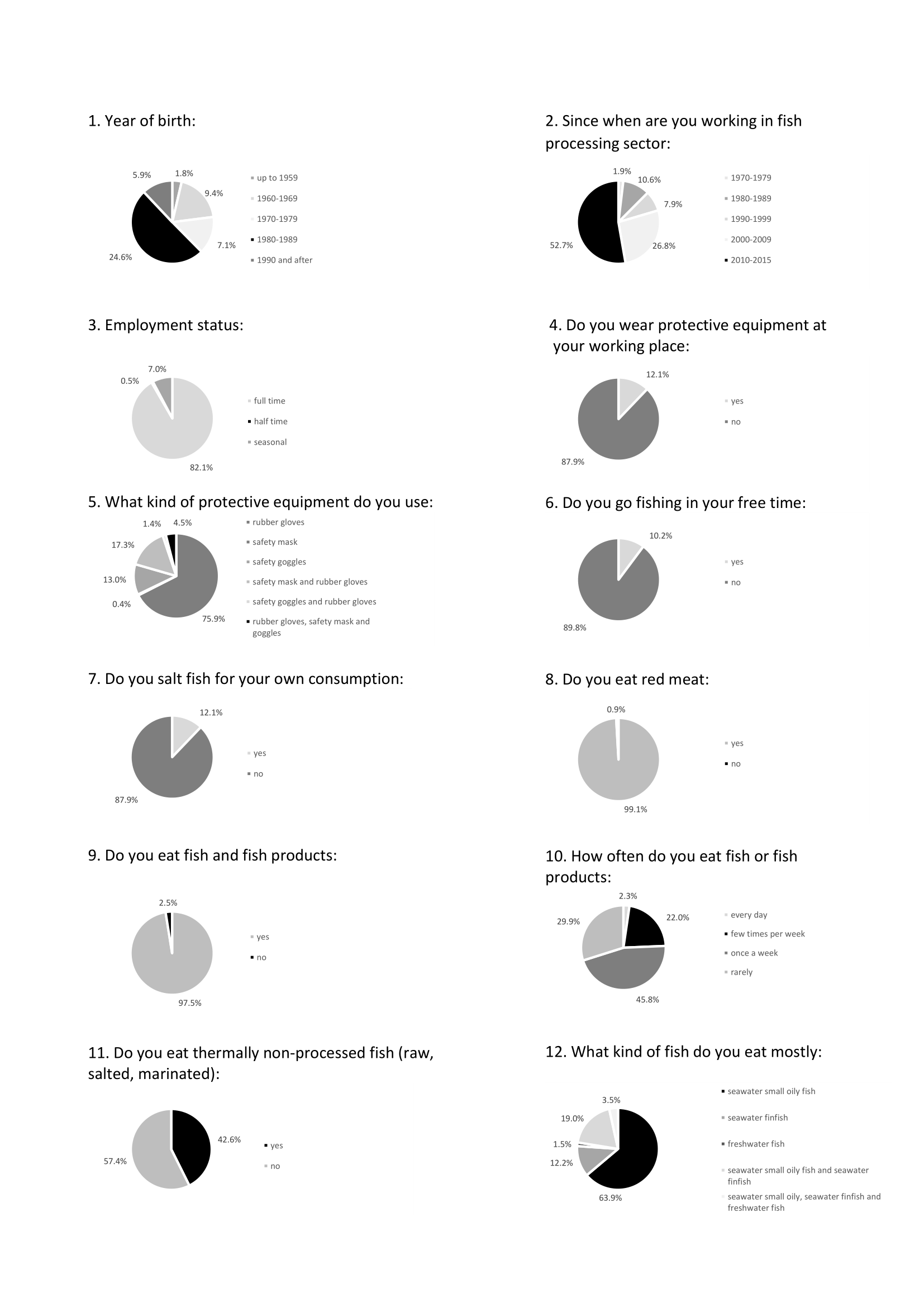

Supplement: S1 Fig — (TIFF) [file pntd.0008038.s007.tiff]

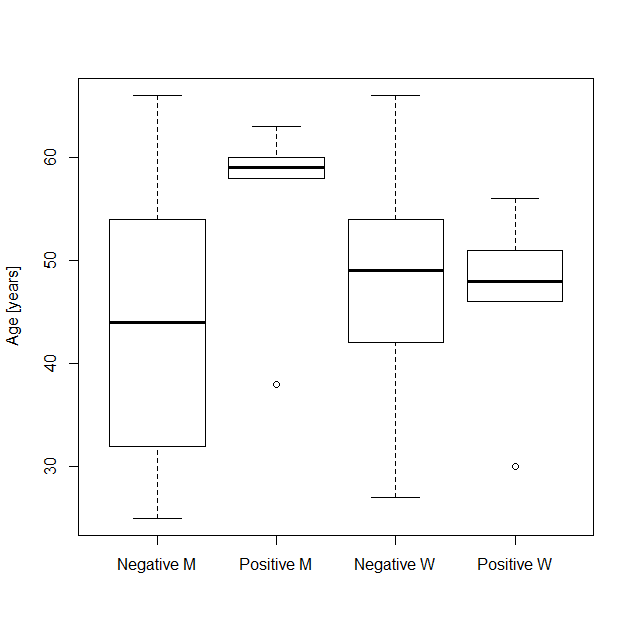

Supplement: S2 Fig — (DOCX) [file pntd.0008038.s008.docx]

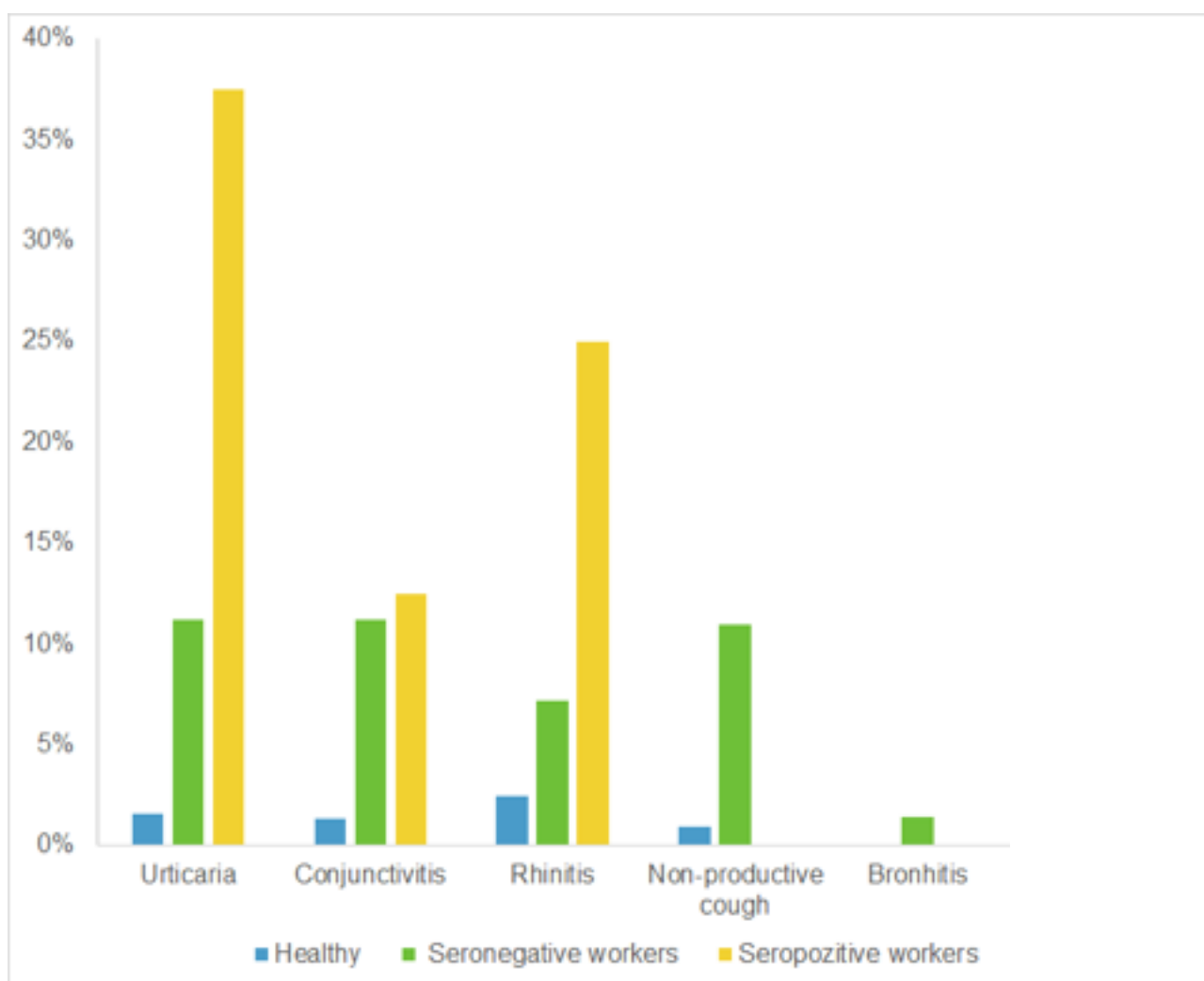

Supplement: S3 Fig — (PDF) [file pntd.0008038.s009.pdf]
